# Multiple Nonfamilial Unilateral Trichoepitheliomas: Report of a Case—Mini Review of the Literature

**DOI:** 10.1155/2019/6821854

**Published:** 2019-07-14

**Authors:** I. Mandekou-Lefaki, G. Theodosiou, F.-S. Delli, D. Oikonomou, M. Papageorgiou

**Affiliations:** ^1^Dermatology, Geniki Kliniki–Euromedica Private Hospital, Thessaloniki, Greece; ^2^Department of Dermatology, Skåne Univeristy of Skåne, Malmö, Sweden; ^3^State Dermatology Clinic, Hospital for Skin and Venereal Diseases, Thessaloniki, Greece; ^4^Plastic Surgery, Private Practice, Thessaloniki, Greece

## Abstract

Trichoepitheliomas are benign skin tumors with follicular differentiation that present most commonly as solitary lesions. They can also present as multiple centrofacial papules due to several mutations in the CYLD gene. Multiple unilateral trichoepitheliomas in a linear or dermatomal distribution may rarely be seen. Herein, we report a case of multiple unilateral trichoepitheliomas on the face of a healthy 34-year-old woman of Caucasian origin.

## 1. Case Report

A 34-year-old woman of Caucasian origin was referred to the outpatient clinic of our department with a rash on the right side of her face. At the age of five years, multiple asymptomatic skin-colored firm papules developed in a unilateral configuration on the right side of her face. The rash was asymptomatic but of cosmetic concern to the patient. The family history was unremarkable. The patient was otherwise healthy.

**Figure 1:**
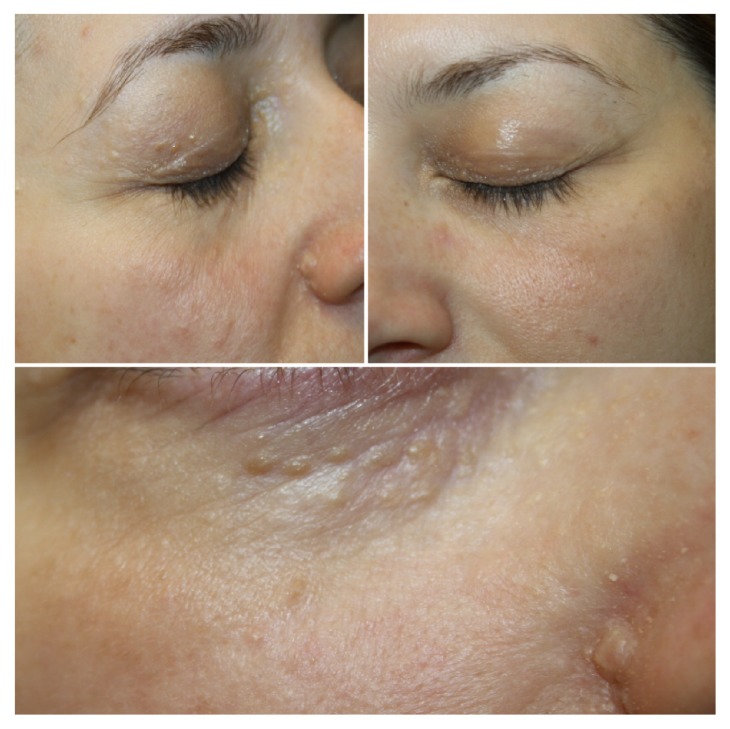
(a) Numerous skin-colored papules on the right side of the patient's face. (b) The left side of the face is free of lesions. (c) Closer view of the right side: multiple rounded whitish and skin-colored papules and nodules symmetrically distributed on the lower eyelid and the perinasal area.

**Figure 2:**
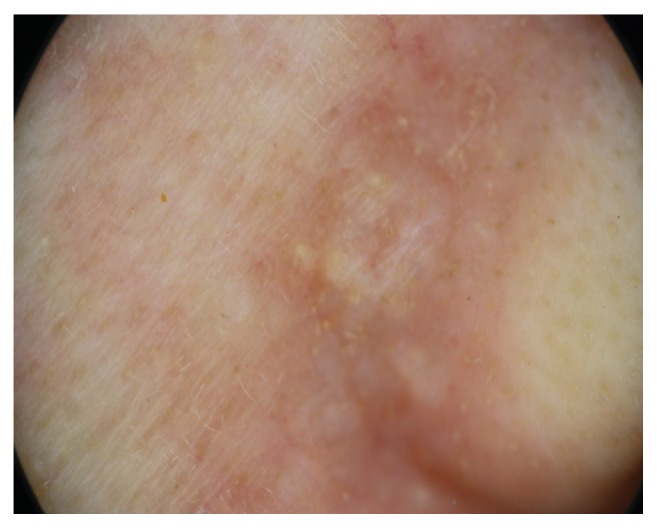
Polarized contact dermatoscopy: on a white-ivory background, bright white lines and yellow to light brown dots and clods.

Physical examination showed skin-colored papules ranging from 2 to 8 mm in size localized on her mid-forehead, the right eyebrow, the eyelids, down the right cheek, and the right nasolabial fold (Figure 1). The lesions were nontender, smooth, and firm. No other skin lesions were noted. Contact polarized dermatoscopy revealed the presence of bright white linear streaks on an ivory-white background as well as yellow and light brown dots and clods (Figure 2)

**Figure 3:**
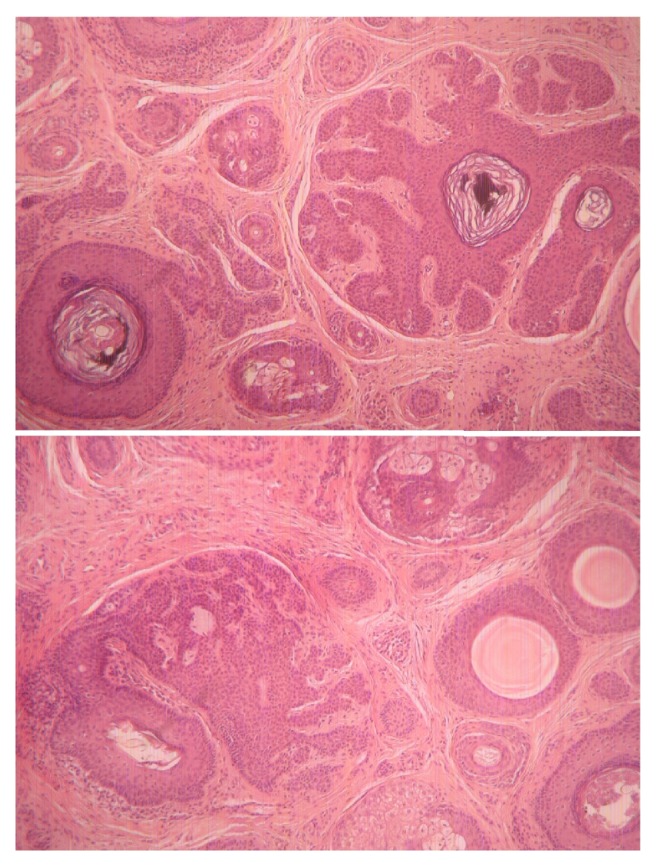
Histopathology: hematoxylin and eosin stain; original magnification x40: multiple horn cysts and aggregations of basaloid keratinocytes surrounded by a fibrous stroma.

Histological examination of a skin biopsy showed a well-demarcated dermal tumor composed of anastomosing lobules of basaloid cells of uniform size arrange in an organoid pattern. The cells were small, with regular nuclei, surrounded by prominent cellular stroma. Small keratinous cysts lined by stratified squamous epithelium were also noted. No connection with the overlying epidermis was noted (Figure 3).

**Figure 4:**
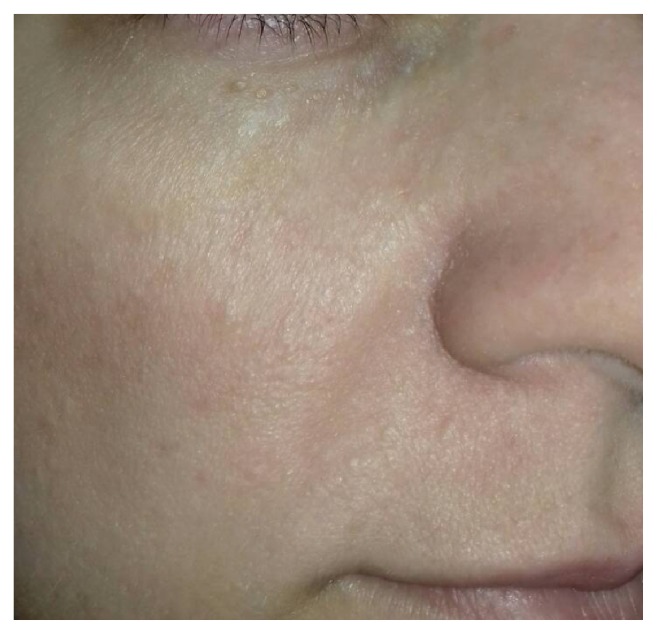
Marked improvement after a 3-month treatment with cream imiquimod 5%.

A lesion in the nasolabial fold was excised in toto and the patient was started on topical treatment with imiquimod 5% cream. The patient applied a thin layer of imiquimod 5% cream prior to normal sleeping hours, left on the lesions for about 8 hours, and then removed by washing the area with mild soap and water, 3 times per week. At the follow-up visit, after three months of treatment with imiquimod 5% cream less than 25% of the lesions at the baseline examination were still present but we also observed a size reduction of the remaining lesions by 1 to 2 mm. The patient was satisfied from aesthetic point of view as she considered that the lesions were less prominent. No side effects were reported during treatment (Figure 4).

## 2. Discussion

Trichoepitheliomas (TE) are benign neoplasms differentiated toward the folliculosebaceous-apocrine germ, occurring mainly in early childhood or adolescence. Their prevalence remains unknown. Trichoepitheliomas increase slowly in size and number throughout life, often causing a significant cosmetic problem [1, 2].

TE are, as a rule, solitary. They present as a solitary skin-colored, round, smooth, firm papule or nodule frequently transversed by telangiectasias on any hair follicle-bearing location, with a predilection for the nose, upper lip, and cheeks. The size can vary from a few millimeters to several centimeters, but most trichoepitheliomas measure not over 1 cm in diameter. Rare presentations include a linear form and a large hemifacial plaque form. The solitary type occurs more frequently in adults [1].

Multiple familial trichoepitheliomas (MFT) usually begin in early childhood or adolescence as multiple round, firm, pink, or skin-colored, sometimes translucent, papules or nodules symmetrically distributed on the face particularly in the perinasal area but can also occur on the trunk and the extremities [2, 3]. MFT have an autosomal dominant mode of inheritance. Various mutations in the CYLD gene, a tumor suppression gene located on chromosome 16q12-q13, have been associated with inherited disorders characterized by multiple adnexal tumors such as trichoepitheliomas, cylindromas, and spiradenomas namely, Brooke-Spiegler syndrome (BSS), multiple familial trichoepithelioma, and familial cylindromatosis [4]. Multiple familial trichoepithelioma and familial cylindromatosis are considered phenotypic variants of BSS.

Multiple trichoepitheliomas have been reported as a rare feature of the Bazex-Dupré-Christol syndrome which is an X-linked dominant disorder characterized by congenital hypotrichosis, follicular atrophoderma, milia, basal cell carcinomas, and hypohidrosis [5, 6]. MFT are often cited feature of Rombo syndrome which is characterized by follicular atrophy, atrophoderma vermiculata, acral cyanosis, milia, hypotrichosis, and basal cell cancers. As a matter of fact, multiple trichoepitheliomas were reported only in one family member from the original case [7]. James Rasmussen described in 1975 a combination of multiple trichoepitheliomas, cylindromas, and milia. Although the term “Rasmussen's syndrome” is still in use, it is nowadays considered a variant of BSS [8].

Rare presentations include linear lesions on the face arranged along the Blaschko lines, and association with ungual fibromas [9–14]. Multiple nonfamilial trichoepitheliomas have also been reported. However, it is unclear whether genetic analyses were performed in these patients [15, 16]. Only a few cases of unilateral multiple trichoepitheliomas have been reported in the literature most of which occurred in dark-skinned individuals [9–11].

Histologically, TE are symmetrical, well-circumscribed nonulcerated dermal neoplasms comprised mostly of follicular germitive cells that are usually arranged in a cribriform pattern. The epithelial cells are deeply basophilic and uniform and usually overlap each other. In addition to the predominant epithelial component, there is also a connective tissue component that resembles that of an embryonic perifollicular sheath. In short, it is abundant highly fibrocystic and constituted mostly of delicate fibrillary collagen. The histological features of the solitary and the multiple variants are identical [17].

Multiple trichoepitheliomas must be differentiated from other adnexal tumors such as eruptive syringomas and the angiofibromas of tuberous sclerosis. The main differential diagnosis for solitary trichoepithelioma is basal cell carcinoma (BCC). Absence of specific follicular stroma, asymmetry, or invasive growth and clefts between epithelial aggregations and the surrounding stroma are clues favoring a diagnosis of BCC. Malignant transformation of trichoepitheliomas is a very rare event, convincingly documented only in a handful of cases [18, 19].

Trichoepitheliomas, as a rule, cannot be differentiated from basal cell cancer solely on specific expression of cytokeratins. A panel of immunohistochemical markers composed of CD10, CK15, CK20, and D2-40 that could facilitate the differentiation trichoepithelioma and basal cell carcinoma has been proposed [20].

Multiple trichoepithelioma on the face present a major cosmetic concern and are typically difficult to treat. The management of trichoepitheliomas is challenging especially in patients with multiple facial lesions. Various treatment modalities have been efficacious in treating trichoepitheliomas, namely, surgery, electrodessication, CO_2_ laser, Erbium–YAG laser, and imiquimod [21–23]. Successful use of topical sirolimus alone or in combination with CO_2_ ablative laser has been also reported [24].

Imiquimod is an immunomodulating agent approved for the treatment of HPV infections, actinic keratoses, and basal cell carcinomas. Imiquimod induced a potent antiviral and antitumor effect by activating the Toll-like receptor 7 (TLR-7) subsequently leading to secretion of interferon-gamma (IFN-*γ*), tumor necrosis factor-a (TNF-a), IL-1a, IL-1b, IL-6, IL-8, IL-12, and GM-CSF. Many studies have tested the effect of imiquimod on several types of skin tumors with mixed results.

In our case, partial response and size reduction to topical therapy with imiquimod were achieved. This is in line with the finding of Alessi et al. who reported two patients with multiple trichoepitheliomas who showed partial response to topical treatment with imiquimod 5% for 32 weeks [25].

In our case, the combination of excision of the largest lesion with topical imiquimod treatment in the smaller lesions brought a satisfactory result for the patient.
